# Spatiotemporal Evolution of Ecosystem Health of China’s Provinces Based on SDGs

**DOI:** 10.3390/ijerph182010569

**Published:** 2021-10-09

**Authors:** Run Zhao, Chaofeng Shao, Rong He

**Affiliations:** 1 College of Environmental Science and Engineering, Nankai University, Tianjin 300350, China; 2120190607@mail.nankai.edu.cn; 2 Sichuan Academy of Environmental Policy and Planning, Chengdu 610041, China; heenhr@163.com

**Keywords:** ecosystem health, ecological scarcity, SDGs, natural capital, artificial capital

## Abstract

In the context of increasing ecological scarcity, maintaining the balance between natural and artificial capital has become a popular research topic in the field of ecosystem health. From the perspective of coordinating natural and artificial capital and maintaining the balance between human systems and the Earth’s ecosystem, the Ecosystem Health Index (EHI) was developed on the basis of the Sustainable Development Goals (SDGs). The EHI consists of the Social Progress Index (SPI), Economic Development Index (EDI), Natural Environment Index (NEI), and a pressure adjustment coefficient. Comprehensive indicator assessment models were used to analyze the spatial and temporal evolution of the EHIs in 30 of China’s provinces from 2013 to 2019. A three-dimensional judgment matrix was used to classify the 30 provinces into four basic types. The results show the following: (1) From 2013 to 2019, the EHIs of all provinces improved to different degrees, with 19 provinces achieving a healthy state. (2) Spatially, the EHI showed some regional aggregation in 2013. Provinces with high EHIs were concentrated in the west, followed by those in the east, and those in the central provinces had the lowest EHIs. However, the differences between regions had narrowed by 2019. (3) The spatial distribution patterns of the NEI and the EDI varied widely, and most provinces did not reach a high level of coordination between natural and artificial capital. (4) The environmental pressure in all provinces, except Liaoning, decreased over time. In some cases, excessive pressure decreased the pressure-adjusted EHI, regardless of the EHI value. (5) According to the results of the ecosystem health classification in each province, the factors that hinder ecosystem health vary from place to place.

## 1. Introduction

Ecosystem health is a characteristic defined by the structural and functional integrity of an ecosystem in the face of disturbances from human activity [1,2]. Early scholars argued that a healthy ecosystem can repair itself despite disturbances, and requires only minimal external support in its management [3]. Other scholars [4] have considered ecosystem health to be the absence of damage or deterioration of the ecosystem organization. Holling et al. [5] added stability, vitality, and sustainability to the criteria for ecosystem health on the basis of the lack of disease. Haskell, Norton, and Costanza [6] defined ecosystem health as a healthy, disease-free system that is stable and sustainable; that is, the system is able to maintain its organizational structure, self-regulate, and recover from stresses over time. Since the initial development of the ecosystem health concept, it has been supplemented and refined by scholars. Mageau [7] argued that a healthy ecosystem supports human communities by providing ecosystem services, such as food, fiber, waste absorption and recycling, drinking water, and clean air. Rapport et al. [8,9] emphasized that the ability to meet reasonable human needs is also an important element of health, in addition to its ecological aspects. Two definitions have evolved, namely, natural ecosystem-centered geocentrism and anthropocentrism, which focuses on the role of system health in the lives of humans and their environment [10]. New advances in ecosystem health assessment methods have been achieved with the enrichment of the meaning of ecosystem health. Hong et al. [11] applied a social accounting matrix, logistic Steele-based land use simulation, and stream ecosystem background conditions as three sub-models for evaluating the health of a stream landscape ecosystem. Van Niekerk et al. [12] evaluated the stresses caused by various socio-ecological processes and analyzed the ecosystem response separately to evaluate the estuarine ecosystem health. The main assessment methods are the indicator species and indicator system approaches [13]. The indicator species method focuses on analyzing environmental changes and assessing the health of ecosystems according to the number of dominant and sensitive species in the community, which is suitable for a single ecosystem and the measurement of a large number of species. The indicator system method combines physics, biology, ecology, economics, and other disciplines to identify suitable comprehensive indicators, which are then used to establish a system of indicators to comprehensively evaluate the health of ecosystems, with the VOR (vitality, organization, and resilience) model being the most commonly used. Assessment techniques based on biodiversity, ecosystem service level, and the comprehensive index of ecosystem health were developed and have been widely used to assess the health status of typical ecosystems, such as forests, rivers, and lakes [14,15,16,17,18]. These studies conducted useful explorations for the sustainable development of ecosystem health and the resource environment at the regional level.

In September 2015, the 2030 Agenda for Sustainable Development [19] was formally adopted in the United Nations Sustainable Development Summit, identifying 17 Sustainable Development Goals (SDGs). The SDGs emphasized the importance of sustainable ecosystem management to ensure the provision of various ecosystem services for future generations. However, the Sustainable Development Goals Report [20] showed insufficient progress on the SDGs related to ecological and environmental areas, deviating from the direction toward sustainable development. Resource scarcity, ecological damage, and the lack of green transition development have become threats to the health of global ecosystems. This phenomenon implies an intergenerational shift in the “scarcity” factors governing socio-economic development; that is, the main scarcity factors that constrain production are shifting from artificial capital to natural capital [21]. A growing conflict exists between the ability of ecosystems to provide limited services to humans and the unlimited demands of human socio-economic development. However, previous studies [22,23,24] on ecosystem health have not addressed the issue of ecological scarcity, with resource scarcity and ecological degradation as the core, and have failed to link ecosystem health with the sustainable development needs of human society from the perspective of coupled human–Earth systems. In addition, previously constructed index systems have mostly relied on the traditional evaluation framework, while lacking an index system that dovetails with the international evaluation framework.

The essence of ecosystem health, the goal of sustainable development, lies in the basic guarantee of providing ecological conditions for sustainable development. Healthy ecosystems are the cornerstone for future sustainable development. Both sustainable development measures and the ecosystem health evaluation system characterize the coupling and driving relationships of human-Earth systems, so the former can provide a reference for the construction of the latter. In this study, we combined the relationship between human and natural systems, considered the connotation of ecosystem health and the 17 SDGs, and built an ecosystem health evaluation system from the perspective of coordinating natural and artificial capital based on SDG assessment. An ecosystem health index (EHI) consisting of three subindexes, namely, the social progress index (SPI), the economic development index (EDI), and the natural environment index (NEI), and a pressure adjustment coefficient (P) was established. This study was conducted in China at the provincial scale to diagnose the effectiveness of current environmental protection actions and provide information support for sustainable ecosystem management.

## 2. Materials and Methods

### 2.1. Evaluation Basis

The concept of “health” was originally used to describe the human body and other animals and plants in a sound physical state [25,26]. The WHO defines health as not only the absence of disease or infirmity, but also the complete physical, psychological, and social state of well-being. On the basis of the relationship between ecosystems and humans, the health definition can be extended to ecosystems. Ecosystem health can be understood as the stability of the capital stock and the sustainability of the capacity to provide ecosystem services from the perspective of the structural function of the ecosystem and its mutually beneficial relationship with humans. Sustainable development is dedicated to searching for the reasonable self-reliance of the socio-economic–natural development system in terms of “development, coordination, and sustainability.” From the ecological aspect, we pursue a reasonable balance between ecosystem health and socio-economic development. The core of sustainable development-oriented ecosystem health addresses the relationship between humans and nature to ensure a balance between society’s demands on and returns to nature; that is, the increase in artificial capital does not presuppose the destruction of and reduction in natural capital. As a source of human welfare, natural ecosystems play an important role in improving the lives of human beings by providing them with the means of production and living; ecological resources are transformed into economic and material wealth to realize the accumulation of artificial capital, which is then invested in social development and regional construction. Natural ecosystems are also the material carriers of human life, providing direct ecological services, such as fresh air, clean water, and good environmental quality [27]. In addition to these desired outputs, the process of transforming natural capital into materials for human consumption has undesirable outputs, such as environmental pollution, with negative impacts on ecosystem health. To ensure the sustainability of ecological services and provide the basic guarantee of natural capital for humans, ecosystems must be managed sustainably.

**Figure 1:**
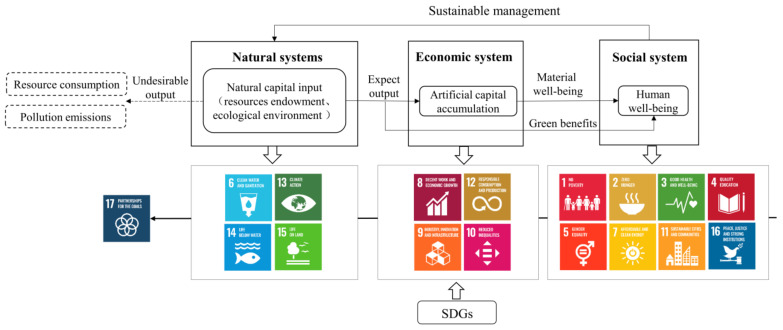
Logical framework of ecosystem health.

With a timeline from 2015 to 2030, the SDGs aim to address the social, economic, and environmental dimensions of development in an integrated manner to harmonize the relationship between humans and nature, and promote the shift of human society toward a sustainable development path [19]. Since the release of “Our Common Future” in 1987, global consensus has been reached on three major aspects that define sustainable development: promoting scientific and technological innovation to overcome the diminishing marginal benefits of growth and provide economic development momentum; maintaining the increase in wealth and sustaining the quality of economic development without sacrificing the ecological environment; and ensuring that institutional construction can increase rational and orderly social management and realize fairness in economic development [28]. The three main elements that influence the health of the Earth’s ecosystems according to the theory of sustainable socio-economic growth and the meaning of ecosystem health are presented in Figure 1, addressing the questions of how natural systems sustainably provide abundant ecological resources, which is a fundamental prerequisite for ecosystem health, and how human systems fully utilize efficient resources to sustain the dynamics of economic development while improving ecosystem health. The ultimate goal of both of these aspects is to achieve the health and well-being of human systems so that the dynamic balance between humans and nature can be effectively maintained and the ecosystem can exist in a stable and healthy condition.

### 2.2. Establishment of Evaluation Index System

**Figure 2:**
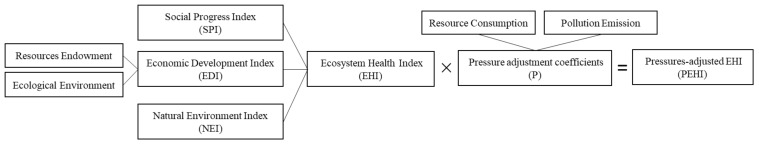
The hierarchical scheme of EHI and PEHI.

From the perspective of the natural–social–economic composite ecosystem, the need to coordinate natural and artificial capital under ecological scarcity is considered in this paper. An evaluation index system of the EHI for benchmarking SDGs was established, including three subindexes and a pressure adjustment coefficient (P), as shown in Figure 2 and Table 1.

#### 2.2.1. Indicators of the SPI

Social progress reflects the systematic level of social construction and SPI comprises eight indicators, including regional urbanization, education, employment, medical care, consumption, social equity, and other aspects.

#### 2.2.2. Indicators of the EDI

Economic growth is evaluated in terms of economic development, industrial structure, and per capita income. Fixed capital is an important factor affecting economic growth. The growth rate of GDP and disposable income are indicators of the level and speed of economic development. The contributions of secondary and tertiary industries to GDP are important indicators of the level and quality of modernization of the national economy. The higher the proportion, the higher the level of economic development. The proportion of R&D expenditure to regional GDP reflects the scale and intensity of R&D activities and the investment in science and technology innovation.

#### 2.2.3. Indicators of the NEI

In this study, the natural environment is divided into two aspects: natural resources and environment. Natural resources are the main source and material basis of production and living, and the environment indicates the quality of the ecological environment, including water, air, soil, and ecological environments. Five indicators were used to reflect resource endowment conditions: energy, water, arable land, forest, and wetland resources. Eight indicators cover water, air, and soil quality and environmental inputs, measuring the quality and sustainability of the ecological environment.

#### 2.2.4. Indicators of P

The above three subindexes are categorized as state quantities. Several pressure indicators also have an impact on ecosystem health, such as pollution emissions and resource consumption. However, these pressures are lagging indicators and provide no clear indication of the current state. They are only able to characterize trends to a certain extent.

This paper draws on the idea of the Planetary pressures-adjusted Human Development Index (PHDI) [29] by using an adjustment factor (P, where a high value implies low pressure). P consists of indicators of resource consumption and pollution emissions (including greenhouse gas emissions). The rate of human resource exploitation must be kept below the rate of resource output, and waste emissions should be kept below the capacity of the environment to absorb and transform waste, which is necessary to prevent the destruction of the biosphere’s ability to produce resources and maintain the services needed for society to survive and thrive. The PEHI is based on the EHI adjusted by P. 

Due to the different levels of economic development and population sizes in different regions, some indicators cannot be directly compared and must be expressed by using the per capita or intensity value. According to the purpose of evaluation, indicators reflecting resource endowment use the per capita value, and some indicators reflecting the level of green development use the intensity value, which better reflects the quality of economic development.

### 2.3. Determination of Indicator Weights and EHI Evaluation Models

To avoid the drawbacks of the subjective weighting method, the entropy weight method [30,31,32] was adopted to calculate the weight of each indicator. Then, the scores of social progress (SPI), economic development (EDI), natural environment (NEI), and pressure adjustment coefficient (P) were calculated for each province from 2013 to 2019 according to the weights. The specific steps and formulas are as follows:

1. The initial indicators are standardized.
  Both positive and negative indicators are expressed as Equation (1) [33,34]:
  (1) $$Y_{ij}=\frac{X_{ij}-X_{j\;wor}}{X_{j\;opt}-X_{j\;wor}}$$
  where *Y_ij_* is the standardized value of *X_ij_*; *X_ij_* is the initial value of indicator *i* in province *j*; and *X_j opt_* and *X_j wor_* are the optimal and worst values of indicator *i*, respectively. The method for determining the threshold is detailed in Section 2.4.
  The normalization matrix is constructed according to the standardized results using Equation (2):
  (2) $$E={\left(Y_{ij}\right)}_{m\times n}=\left[\begin{array}{cccc} X_{11} & X_{12} & \ldots & X_{1n}\\ X_{21} & X_{22} & \ldots & X_{2n}\\ X_{m1} & X_{m2} & \ldots & X_{mn} \end{array}\right]\;$$
  where *m* is the number of evaluation indices, and *n* is the number of evaluation objects.
2. The entropy value ($E_{i}$) and difference coefficient ($H_{i})\;\mathrm{of}\;\mathrm{indicator}\;i$ are calculated as Equations (3) and (4):
  (3) $$E_{i}=\frac{1}{\ln m}\overset{m}{\underset{i=1}{\sum }}\frac{X_{ij}}{\sum_{i=1}^{m}X_{ij}}\ln\frac{X_{ij}}{\sum_{i=1}^{m}X_{ij}}$$
  (4) $$H_{i}=1-E_{i}\;$$
3. Weight $\omega_{i}$ is calculated as Equation (5):
  (5) $$\omega_{i}=\frac{H_{i}}{n-\sum_{i=1}^{n}H_{i}};\left(0\leq \omega_{i}\leq 1\right),\overset{n}{\underset{i=1}{\sum }}\omega_{i}=1,$$
  where $\omega_{i}\;$is the weight of indicator *i*.
4. According to the corresponding weights, the score (*U_k_*) of each subindex (including P) is derived as Equation (6):
  (6) $$U_{k}=\sum_{i=1}^{n}Y_{ij}\omega_{i}$$
5. The *EHI* score is calculated as Equation (7):
  (7) $$EHI=\alpha U_{1}+\beta U_{2}+\gamma U_{3}$$
  where *α*, *β*, and *γ* are undetermined parameters. As the three subsystems can influence and complement each other and are equally important in the larger system, each takes 1/3 of the weight.
6. The *PEHI* after adjustment [29] is calculated using Equation (8):
  (8) PEHI=P×EHI,
  where the *PEHI* represents the *EHI* adjusted by pressure. The value of *P* is within the range of [0, 1], and a high value means low pressure.

### 2.4. Determination of the Threshold Value

#### 2.4.1. Discussion of Indicator Characteristics

Although the most common evaluation indicators are positive and negative indicators, moderate indicators also exist. These indicators have a tolerance range, which should neither be extremely large nor small, such as the urbanization rate, the number of medical practitioners (assistants) per 1000 people, and the intensity of land development, all of which are used in our study.

An extremely low urbanization rate indicates that economic growth in the urbanization process has not been fully realized. Conversely, an extremely high value indicates the excessive expansion of cities and towns, and the large-scale aggregation of the population in cities and towns will cause a series of problems. For example, the number of available jobs cannot meet the demand, and the expansion of cities occupies a large amount of farmland, which poses many hidden dangers to China as a major agricultural country. In serious cases, providing sufficient food to people becomes a severe problem. In this paper, the optimal level of developed countries is set to 80%, and an urbanization rate ranging from 0 to 80% is regarded as a positive indicator (0 is the worst value and 80% is the optimal value), which can be directly used in standardized calculations. Once the value exceeds 80%, the metric is normalized to 0.

The intensity of land development follows the same approach as that of the urbanization rate. When the intensity of land development exceeds a certain limit, people’s living environment will be threatened. According to “Outline of National Land Planning (2016–2030)”, China’s land development intensity will be controlled within 4.62% by 2030. Therefore, in this study, 4.62% is considered the optimal value, as stipulated by China, and the interval between 0 and 4.62% is regarded as a positive indicator. 

An extremely small number of medical practitioners (assistants) indicates a lack of medical resources, while an extremely large number will result in the waste of medical resources. However, the screening of all the data collected during the study period revealed that the maximum value of this indicator among all provinces was 5.85, which does not reach the level of medical resource waste. Therefore, this indicator is regarded as a positive indicator.

#### 2.4.2. Principles for Determining Thresholds

The five-step decision tree method in the Sustainable Development Report [20] was used to determine the optimal and worst values of the indicators after adjustment, considering the actual situation in China. The specific methods are as follows:

1. The indicators that are explicitly included in the SDGs with absolute thresholds, such as the urban registered unemployment rate and the Gini coefficient, are directly adopted. The absolute value is regarded as the value of the indicator, whose optimal value is 0.
2. For the indicators that are not explicitly required in the SDGs but have a desirable accepted value, such as the years of schooling per capita, the accepted value is selected as the optimal value.
3. Some indicators have stipulated limits in China, such as the ratio of nature reserves to jurisdictional areas; in this study, the optimal value of this indicator is set to 15% in counties, cities, and provinces in China. The optimal value for fertilizer application intensity is 250 kg/ha.
4. For the indicators not included in the three scenarios above, the average of the three best/worst performing provinces is always selected as the best/worst value.

### 2.5. Classification of Health Levels

A comprehensive index was used to classify ecosystem health status in previous studies [35]. As such a classification can be easily influenced by a subsystem that scores extremely high or low and obscures the original weak (superior) term, we classified the ecosystem health of the subsystem into three levels [23,36] on the basis of the calculated subindexes of social progress, economic development, and natural environment according to the Natural Breaks method of thematic classification in ArcGIS (Table 2). It is important to emphasize that ecosystem health is a relative concept as it is defined according to the reference values of indicators, and the Natural Breaks method of thematic classification, which is based on the natural grouping inherent in the data, identifies the classification intervals. Thus, the breaks of the three levels are “local”, not “global”; in other contexts and with other data, the values of the breaks would be different.

The ecosystem health status of each province was identified by establishing a three-dimensional assessment matrix [37] based on the health level of each subsystem (Table 3), similar to the identification methods of health status adopted by other scholars [23]. The rules are as follows:

◆ If all three subindexes have values at the “healthy” level, that is, H = 3, the province has a comprehensive health status, that is, social–economic–natural health.
◆ If all three subindexes have values at the “subhealthy” level, that is, S = 3, the province has a subhealth status, that is, social–economic–natural subhealth.
◆ If one or more of the three subindexes have values at the “disease” level, that is, D ≥ 1, the province has the corresponding disease status based on the “one-vote veto system”.
◆ If one of the three subindexes has a value at the “healthy” level, that is, H = 1 and D = 0, the province has a single health status, such as natural health or economic health.
◆ If two of the three subindexes have values at the “healthy” level, that is, H = 2 and D = 0, the province has a compound health status, such as economic-natural health or social–economic health.

Social–economic–natural health is defined as the comprehensive health status. Social–economic, social–natural, and economic–natural health are compound health statuses, and social, economic, and natural health are single health statuses. Social–economic–natural subhealth is the subhealth status, and social, economic, and natural disease are single disease statuses. Social–economic, social–natural, and economic–natural disease are compound disease statuses, and social–economic–natural disease is defined as the comprehensive disease status.

### 2.6. Data Source

In 2011, the 7th National Environmental Protection Congress of China clarified the new principle of “Development in protection and protection in development,” and China began to explore a new path of green, high-quality, and sustainable development. Since then, the laws and regulations related to ecological and environmental protection have been updated to improve the health of the ecosystem. The year 2013 was an important landmark year for China in its commitment to fighting environmental pollution and starting a new path toward harmonizing the development of human and natural environments. Thus, 2013 was used as the starting point of the study period. The research endpoint is 2019, as the latest data available are from 2019. In the selection of sample regions, Tibet, Hong Kong, Macau, and Taiwan were excluded due to difficulties in obtaining data. The data of 30 other provinces (municipalities and autonomous regions) in China from 2013 to 2019 were obtained from regional statistical yearbooks, local government websites, national environmental statistical bulletins, the China Environmental Statistical Yearbook, the China Statistical Yearbook, and the China Energy Statistical Yearbook. Individual missing data were calculated using the average, exponential trend extrapolation, and interpolation methods.

## 3. Results and Discussion

### 3.1. Analysis of EHI Temporal Characteristics 

#### 3.1.1. Analysis of Country-Level Temporal Characteristics 

**Figure 3:**
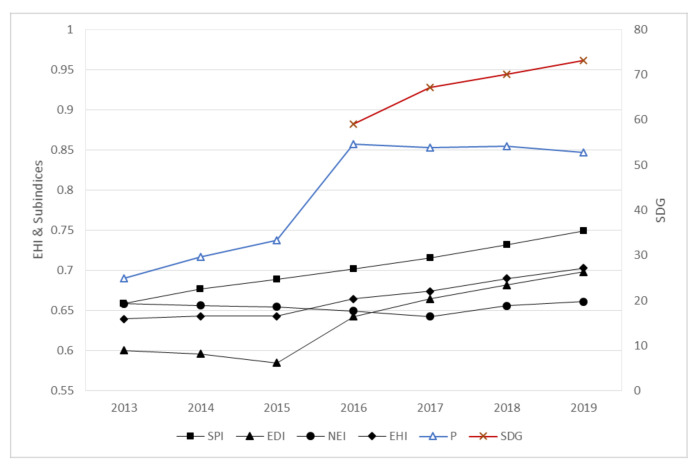
Trends in China’s ecosystem health index and subindexes, 2013–2019.

Since 2013, China has issued a series of major policy decisions about environmental protection, including the revision of laws and emission limits for the prevention and control of water, air, and soil pollution in the ecological and environmental field. The results of this study’s evaluation clearly indicate that this series of initiatives has achieved good results at the national level (Figure 3): China’s EHI increased from 0.6395 in 2013 to 0.7029 in 2019, showing a positive trend, which is consistent with China’s performance in the SDGs evaluation (the SDGs evaluation started in 2016). Each subindex shows different degrees of improvement. The SPI increased from 0.6590 in 2013 to 0.7494 in 2019, the EDI improved from 0.6006 to 0.6981, and the NEI was stable, fluctuating in a small range above and below 0.66. The improvement of the EDI and SPI indicates that the capacity of natural systems to supply services to human society improved, as natural capital was not reduced or lost. The coordinated development of artificial and natural capital confirms the effectiveness of the initiatives introduced in China. The pressure also decreased annually, with P increasing from 0.690 to 0.847, implying that various human production activities exerted low negative pressure on the natural environment and became less of an obstacle to the achievement of sustainable development.

Figure 3 indicates a brief decline in the EDI between 2013 and 2015 as China’s economy was in full transition to the new normal, and the real estate ended its 15-year-long boom period and entered an adjustment period, leading to a significant increase in downward pressure on the economy during that period. China’s EDI returned to a positive trajectory after 2015 and had improved to 0.698 by 2019 due to the acceleration of institutional reforms, stock adjustment, and incremental optimization in China. The EHI trend is basically the same as the EDI trend, indicating that economic development determines the direction of ecosystem health in China’s current stage of development. At present, China’s GDP per capita is slightly over USD 10,000, which is roughly equivalent to only 90% of the world average, with a gap of approximately 20% from the standard of high-income countries. Thus, achieving high-quality economic development remains a challenge. However, extremely high economic growth may lead to the “pollution for growth” of unrestrained development, thereby requiring green transformation development to build a modern society in which people and nature live together in harmony.

#### 3.1.2. Analysis of Provincial Temporal Characteristics

At the provincial level (Table 4), all provinces in the country show an overall increasing EHI trend, with all of them improving to varying degrees in 2019 compared to 2013. The EHI ranges in 2013 and 2019 were 0.5351–0.7411 and 0.6116–0.7627, respectively. Henan exhibited the largest improvement, increasing from 0.5368 in 2013 to 0.6508 in 2019, with a growth rate of 21.23%. From the subindex scores, the increase is mainly driven by the significant improvement of the EDI and the NEI. Specifically, Henan adhered to the development concept of ecological priority and mutual promotion of ecology and the economy and has taken many initiatives to promote the integration of ecological construction and economic and social development.

Anhui, Fujian, Jiangxi, Hubei, Hunan, Guangdong, and Ningxia exhibited a rising trend for 7 consecutive years. The other provinces did not maintain a continuous rising trend, but the fluctuation range was small, and the rise or fall did not exceed 0.06. The largest increase in the EHI occurred in Chongqing between 2018 and 2019, during which it increased by 0.055. Among specific indicators, Chongqing performed better than the other provinces in ecological protection and governance, resource consumption intensity, and resource use efficiency. The largest decline in the EHI occurred in Liaoning between 2013 and 2014, during which it decreased by 0.024, due to a decline in both the EDI and the NEI. Liaoning had a serious problem of insufficient investment in environmental protection during this period.

### 3.2. Analysis of EHI Spatial Characteristics 

**Figure 4:**
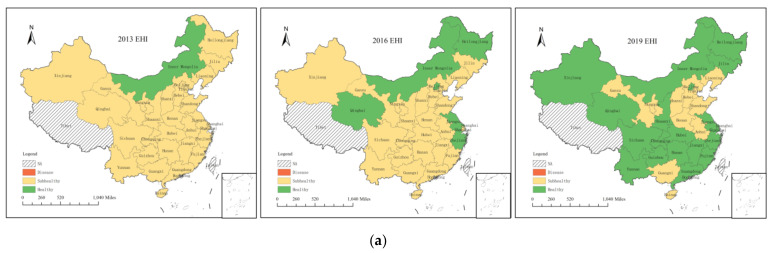

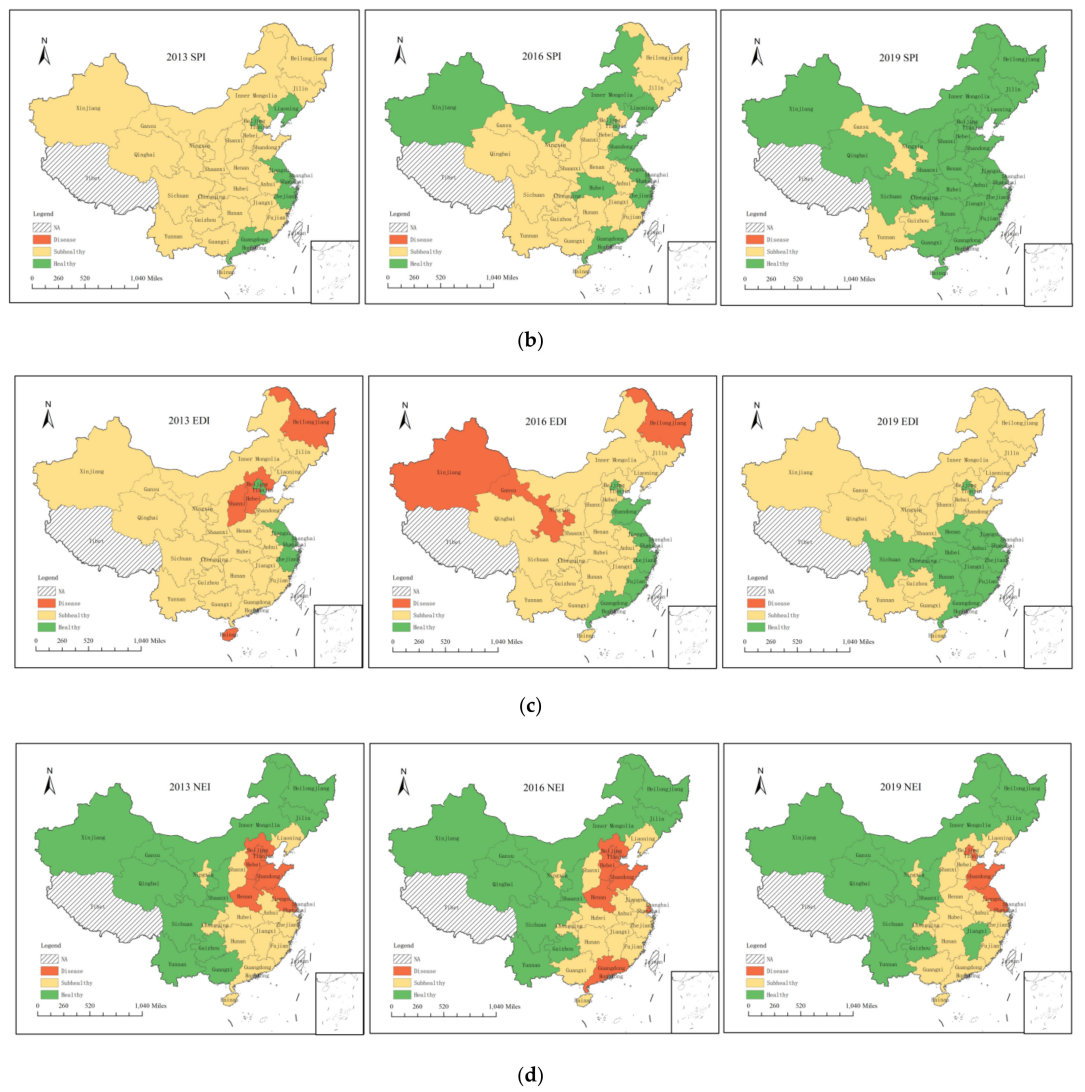
Distribution of EHI and subindexes in 30 provinces. (**a**) EHI; (**b**) SPI; (**c**) EDI; (**d**) NEI.

The characteristics of the spatial pattern were analyzed at three different points in time (i.e., 2013, 2016, and 2019). China can be divided into three major regions: eastern, central, and western. The eastern region includes the 13 provinces (cities) of Heilongjiang, Jilin, Liaoning, Beijing, Hebei, Tianjin, Fujian, Jiangsu, Shandong, Shanghai, Zhejiang, Guangdong, and Hainan; the central region includes the 6 provinces (cities) of Shanxi, Anhui, Jiangxi, Henan, Hubei, and Hunan, and the western region includes the 12 provinces (cities) of Inner Mongolia, Guangxi, Gansu, Ningxia, Qinghai, Shaanxi, Xinjiang, Guizhou, Sichuan, Tibet, Yunnan, and Chongqing. The EHI has a degree of spatial aggregation (Table 4, Figure 4a). In 2013, provinces with high EHIs were concentrated in the western region, followed by those in the eastern provinces, and the central provinces, such as Anhui, Henan, and Shanxi, had poor EHIs. However, through the implementation of the strategy of the rise of Central China, the ecosystem health improved with the commodity food base, important energy sources, and raw material base. By 2019, 19 provinces had transitioned to a healthy state, and the differences between regions gradually narrowed.

In terms of the SPI (Figure 4b), all provinces had good development bases and were already above the subhealthy level in 2013, with the eastern provinces of Beijing, Tianjin, Jiangsu, Zhejiang, and Guangdong performing predominantly at the healthy level. The other provinces transitioned to a healthy state over time. The remote areas of Gansu, Yunnan, and Guizhou, which are located in southwestern and central-western China (mostly in mountainous and basin landscapes with inconvenient transportation and therefore a relatively low level of social development), remained in a subhealthy state, requiring urgent improvement in the services that they can provide for the well-being of their residents.

The EDI (Figure 4c) of the provinces fluctuated considerably. The EDIs of Beijing, Tianjin, Jiangsu, and Zhejiang were at healthy levels in 2013, with a good economic foundation. In contrast, the EDIs of Hebei, Shanxi, Heilongjiang, and Hainan were at the disease level, with relatively low economic development. In 2016, Xinjiang and Gansu, which are located in northwest China, transitioned to a disease state. The terrains of these two provinces are mostly mountains and basins, and the transportation was underdeveloped. Their economic development was relatively low as it was mostly based on agriculture and animal husbandry. The southeast coast transitioned to a healthy state during this period and drove the economic development of the surrounding provinces. Provinces in a good economic state in 2019 are clustered around Beijing and Hubei. Owing to the focus of the western development strategy, the economic situation in the northwest also improved, with no provinces having an EDI in the disease category.

The NEI showed an opposite spatial distribution pattern to the EDI and did not greatly change over the seven study years (Figure 4d), indicating that most of the provinces failed to achieve coordination between natural and artificial capital. The NEIs of the western and northeastern regions were high and remained healthy. Most mid-western and northeastern regions have been blessed with natural resources, such as minerals, energy, and biological resources. Their economy was underdeveloped, making their ecological environment better protected and less damaged than those of the other provinces. The NEIs of the other regions were at the subhealthy or disease level. The NEIs of the provinces centered on Beijing, Tianjin, and Hebei were at the disease level. As the capital of China, Beijing had a high level of economic and social development (Figure 4b,c) but had notable shortcomings in the natural environment. First, the urbanization rate and the population density were high, and the per capita resource endowment was insufficient. Second, the environmental quality and the ecological condition were unsatisfactory. The eastern coastal provinces had a low level of resources due to the low importance placed on resources. As developed regions in China, these provinces had a high level of economic development. They can provide a solid economic foundation for promoting the upgrade of the energy consumption structure, green transformation development, and ecological environment construction. However, the current situation indicates that this ideal state has not yet been reached.

### 3.3. Classification of Provincial Ecosystem Health Level

**Figure 5:**
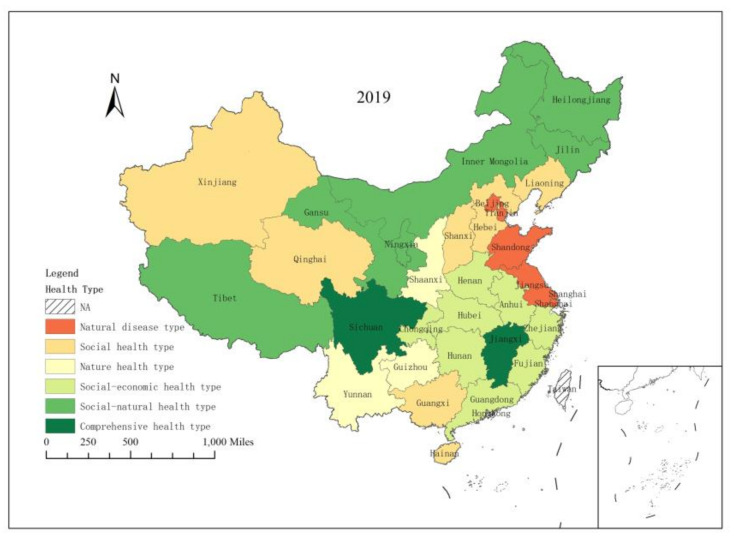
Spatial distribution of ecosystem health classifications in 30 provinces, 2019.

According to the identification rules in 2.5, the 30 provinces in 2019 were classified into four basic types and six subtypes (Figure 5). Two provinces, namely, Jiangxi and Sichuan, achieved comprehensive ecosystem health. Inner Mongolia, Jilin, Heilongjiang, Shaanxi, Qinghai, and Xinjiang were characterized by social–natural health, while Zhejiang, Anhui, Fujian, Henan, Hubei, Hunan, Guangdong, and Chongqing were classified at the social–economic health level. Hebei, Shanxi, Liaoning, Guangxi, Hainan, and Ningxia were socially healthy. Guizhou, Yunnan, and Gansu were at the natural health level. Beijing, Tianjin, Shanghai, Jiangsu, and Shandong were characterized by natural disease. From the classification results, we can conclude that the provincial ecosystem health categories are generally spatially clustered, and some provincial ecosystem health classes have high consistency and local clustering. This classification provides a policy reference for the regional improvement of ecosystem health.

### 3.4. Pressure-Adjusted EHI (PEHI)

The PEHI is obtained according to Equation (8) in 2.3. If a region is not subjected to pressure from pollution emissions and resource consumption, then its PEHI and EHI will be equal. According to Table 5, the environmental pressure improved to varying degrees in nearly all provinces (a large P indicates low pressure). From 2013 to 2019, the environmental pressure was low in the southern provinces of Chongqing, Yunnan, Fujian, Jiangxi, Hunan, and Sichuan, while it was high in the northern provinces of Ningxia, Shanxi, Liaoning, Inner Mongolia, and Xinjiang, especially Liaoning, where pressure was higher in 2019 compared to 2013. This result indicates that the south had better control over resource consumption and pollution emissions than the north. Shanxi, which accounts for 2% of the country’s land area but retains more than 20% of the country’s coal reserves, had the poorest performance of the provinces. The past intensive development model greatly increased Shanxi’s resource consumption intensity, resulting in serious pollution emission problems. However, the environmental pressures faced by the provinces eased with the advancement of the energy revolution and the development of high-quality transformation in the energy industry. Given the complexity of environmental problems, Liaoning failed to maintain its trend of mitigating environmental pressure in 2019 and experienced a temporary decline. Industrial restructuring, environmental pollution treatment, environmental policy establishment, and the promotion of ecological environment construction should be carried out.

**Figure 6:**
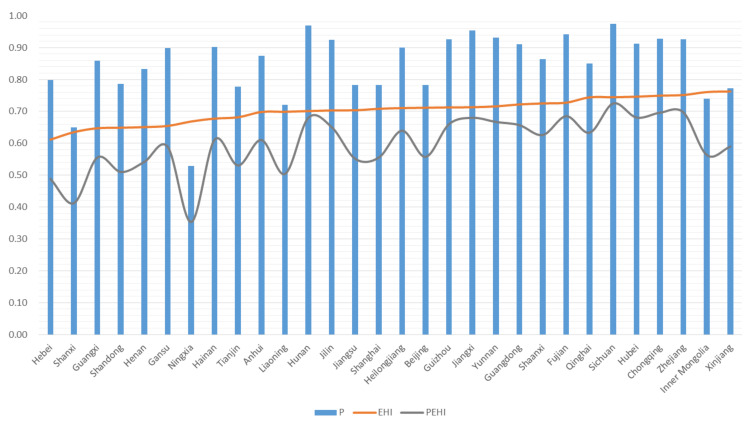
P, EHI, and PEHI of 30 provinces, 2019.

Taking 2019 as an example, the effect of P on the EHI was explored. As shown in Figure 6, extreme pressure (small P value) decreased the final PEHI in some cases, such as in Ningxia, Inner Mongolia, and Xinjiang provinces, regardless of the EHI value. Although China’s provinces had made efforts in green development and pollution prevention, the EHI was still influenced by environmental pressures. This phenomenon can be attributed to the leapfrog development of China’s economy and the complexity of environmental problems caused by spatial and temporal compression, which had multiple effects leading to relatively high pressure on China’s ecological environment and the serious situation around the world. The P of Sichuan was close to 1, indicating that the environmental pressure was low and the PEHI was very close to the EHI. However, except for Sichuan, the PEHIs of other provinces with healthy EHIs (EHI > 0.7) transitioned to subhealthy after the pressure adjustment. The subhealthy EHI decreased to the disease level after pressure adjustment in three provinces: Hebei, Shanxi, and Ningxia. Excessive environmental pressure can lead to the deterioration of natural ecosystems and reduce ecosystem services and functions, which may reduce the social and economic benefits and ultimately affect human well-being. This result indicates that immediate and aggressive initiatives must be implemented to reduce the tremendous pressures on the natural environment caused by human activities. Otherwise, the level of ecosystem health will stagnate.

## 4. Conclusions

In this study, an EHI system was developed for Chinese provincial units based on the SDGs, consisting of the SPI, the EDI, the NEI, and P. Then, we studied the spatial and temporal variation in the EHI in 30 of China’s provinces from 2013 to 2019. The 30 provinces were classified into four basic types based on the three subindexes using a three-dimensional judgment matrix. The main findings are as follows:

(1) In terms of the time series, the overall level of provincial ecosystem health in China was on the rise between 2013 and 2019. The EHIs of all 30 provinces improved to varying degrees, driving the national EHI from 0.6395 in 2013 to 0.7029 in 2019. This trend indicates that the actions taken since 2013 to protect the ecological environment have effectively decreased the conflict between socio-economic development and ecosystem protection and promoted the coordinated development of the human and natural environments.
(2) Spatially, the EHI showed certain regional aggregation at the beginning of the study period. The provinces with high EHIs were concentrated in the western regions, followed by the eastern provinces, and the central provinces had the lowest levels. The differences between regions had narrowed by 2019. In terms of the subindexes, the spatial distribution patterns of the NEI and the EDI differed greatly, and natural and artificial capital did not reach a high level of coordination in most of the provinces.
(3) The environmental pressure was mitigated to varying degrees in all provinces from 2013 to 2019, except in Liaoning. In some cases, excessive pressure decreased the PEHI, regardless of the EHI value. The southern provinces had low environmental stress, while the northern provinces had high stress. There is a distinct spatial distribution of environmental stress.
(4) According to a three-dimensional judgment matrix, the classification of ecosystem health in each province was determined, and potential countermeasures were identified. Each region should focus on its specific characteristics and advantages and clarify the main and secondary aspects to achieve key breakthroughs in certain areas and comprehensively improve regional ecosystem health. Provinces in the social–natural health class had serious deficiencies in R&D investment and low proportions of secondary and tertiary industries. Actions should be taken to improve the innovation of the ecological system and build a market with a diversified science and technology investment mechanism. Meanwhile, effort should be put into realizing the high-quality development of secondary and tertiary industries and continuously optimizing the industrial structure, combined with national industrial policies. Provinces in the social–economic health class should support the development of environmental protection construction with strong capital, advanced technology, and improved systems based on a good economic foundation. These provinces should strengthen the government’s function in the protection of the ecological environment, increase the investment of funds and human resources in ecological environment construction, strengthen the monitoring of the ecological environment, and carry out comprehensive macro-control of regional economic development and environmental protection. Provinces in the social health class shoulder the burden of the dual transformation of economic–social development and protection of the ecological environment. They should strengthen their cooperation with the capital forces of domestic provinces, change their mode of economic development, and optimize their economic structure. They should also maintain their current achievements in protecting the ecological environment and explore the establishment of a scientific and complete system of ecological environment protection. Provinces in the natural health class have healthy natural environmental systems and a low index of economic development. On the one hand, economic development should be continuously accelerated to provide solid support for the overall improvement of regional ecosystem health. On the other hand, the existing advantages should be consolidated, ecological advantages should be fully exploited, and the counter-effect of environmental regulations on the negative consequences of economic development must be enhanced to realize the simultaneous growth of artificial and natural capital. Provinces with developed economies that are grouped into the natural disease class should be guided in the transformation of economic development dynamics through environmental regulations to promote the optimization of industrial structure and green development, and enterprises should be encouraged to intensively implement the concept of green development. Further research and development and promotion of new products, new technologies, and new business models for pollution prevention, green energy, energy saving, and emission reduction should be conducted to realize the gradual replacement of the traditional unrestrained growth model with a green and innovative economic development model.

## Figures and Tables

**Table 1:** Index system of ecosystem health based on SDGs.

Subindex		No.	Indicators	Indicator Character	Benchmark
Social progress		A1	Urbanization rate	○	SDG11
		A2	Average educational year	+	SDG4
		A3	Life expectancy in the population	+	SDG3
		A4	Urban registered unemployment rate	−	SDG8
		A5	Town Engel coefficient	−	SDG1
		A6	Rural Engel coefficient	−	SDG1
		A7	Gini coefficient	−	SDG10
		A8	Per capita household consumption expenditure	+	SDG8
		A9	Number of medical practitioners (assistants) per 1000 people	○	SDG3
Economic development		B1	Fixed capital stock per capita	+	/
		B2	Per capita GDP growth rate	+	SDG8
		B3	Per capita disposable income	+	SDG8
		B4	Proportion of secondary industry in GDP	+	SDG9
		B5	Proportion of tertiary industry in GDP	+	SDG9
		B6	Proportion of R&D expenditure in GDP of the region	+	SDG9
Natural environment	Resource endowment	C1	Per capita reserves of energy resources	+	SDG7
		C2	Per capita water resources	+	SDG6
		C3	Per capita cultivated area	+	/
		C4	Per capita forest stock	+	SDG15
		C5	Per capita wetland area	+	SDG15
	Ecological environment	C6	Proportion of investment in ecological and environmental protection in GDP	+	SDG6, SDG15
		C7	Forest coverage rate	+	SDG15
		C8	Proportion of protected natural area in area under jurisdiction	+	SDG15
		C9	The surface water reaches or is better than the proportion of class ⅲ water body	+	SDG6
		C10	Proportion of days with good air quality	+	SDG11
		C11	Comprehensive utilization rate of industrial solid waste	+	SDG12
		C12	Intensity of fertilizer application	−	SDG12
		C13	Intensity of pesticide application	−	SDG12
Pressure	Resource consumption	P1	Elasticity coefficient of energy consumption	−	SDG7
		P2	Utilization rate of water resources development	−	SDG6
		P3	Intensity of land development	○	SDG12
	Pollution emissions	P4	Emission intensity of ammonia nitrogen	−	SDG12
		P5	COD emission intensity	−	SDG12
		P6	Nitrogen oxide emission intensity	−	SDG12
		P7	SO_2_ emission intensity	−	SDG12
		P8	Solid waste generated per unit of GDP	−	SDG11
		P9	Greenhouse gas emission intensity	−	SDG13

+, −, and ○ indicate positive, negative, and moderate indicators, respectively.

**Table 2:** Subsystem health classification.

Health Levels	Score	Health Status
Level 1	0.7−1.0	Healthy
Level 2	0.5−0.7	Subhealthy
Level 3	0−0.5	Disease

**Table 3:** Health classification when the social subsystem has a “healthy”, “subhealthy” and “disease” status.

Economic Subsystem Health Levels	Natural Subsystem Health Levels		
	Healthy	Subhealthy	Disease
“healthy” status			
Healthy	Social–economic–natural health	Social–economic health	Natural disease
Subhealthy	Social–natural health	Social health	Natural disease
Disease	Economic disease	Economic disease	Economic–natural disease
“subhealthy” status			
Healthy	Economic–natural health	Economic health	Natural disease
Subhealthy	Natural health	Social–economic–natural subhealth	Natural disease
Disease	Economic disease	Economic disease	Economic–natural disease
“disease” status			
Healthy	Social disease	Social disease	Social–natural disease
Subhealthy	Social disease	Social disease	Social–natural disease
Disease	Social–economic disease	Social–economic disease	Social–economic–natural disease

**Table 4:** EHIs of 30 provinces in China, 2013–2019.

Region	2013	2014	2015	2016	2017	2018	2019	Average Value
Beijing	0.6940	0.6798	0.6851	0.7022	0.6935	0.6941	0.7116	0.6944
Tianjin	0.6673	0.6675	0.6647	0.6820	0.6696	0.6927	0.6817	0.6751
Hebei	0.5351	0.5315	0.5441	0.5801	0.5903	0.6126	0.6116	0.5722
Shanxi	0.5935	0.5853	0.5893	0.6001	0.6541	0.6474	0.6350	0.6150
Inner Mongolia	0.7411	0.7470	0.7297	0.7329	0.7270	0.7760	0.7608	0.7449
Liaoning	0.6740	0.6500	0.6348	0.6532	0.6842	0.7213	0.6989	0.6738
Jilin	0.6875	0.6685	0.6607	0.6894	0.6757	0.6902	0.7032	0.6822
Heilongjiang	0.6954	0.6929	0.6837	0.7040	0.7036	0.7120	0.7105	0.7003
Shanghai	0.6682	0.6759	0.6703	0.6784	0.6647	0.6779	0.7084	0.6777
Jiangsu	0.6441	0.6594	0.6762	0.7083	0.7105	0.7019	0.7036	0.6863
Zhejiang	0.6685	0.6869	0.7038	0.7231	0.7072	0.7138	0.7515	0.7078
Anhui	0.5971	0.6054	0.6088	0.6503	0.6538	0.6713	0.6983	0.6407
Fujian	0.6521	0.6583	0.6665	0.6800	0.6955	0.7037	0.7276	0.6834
Jiangxi	0.6105	0.6178	0.6157	0.6412	0.6470	0.6753	0.7132	0.6458
Shandong	0.6042	0.6090	0.6126	0.6343	0.6439	0.6587	0.6490	0.6302
Henan	0.5368	0.5552	0.5531	0.5843	0.6143	0.6195	0.6508	0.5877
Hubei	0.6185	0.6449	0.6487	0.6900	0.6961	0.7210	0.7462	0.6808
Hunan	0.6040	0.6117	0.6241	0.6338	0.6443	0.6650	0.7012	0.6406
Guangdong	0.6374	0.6450	0.6560	0.6831	0.6877	0.6993	0.7221	0.6758
Guangxi	0.6127	0.6135	0.6244	0.6300	0.6145	0.6595	0.6473	0.6288
Hainan	0.5867	0.6056	0.5994	0.6135	0.6348	0.6569	0.6776	0.6249
Chongqing	0.6349	0.6604	0.6558	0.6858	0.6976	0.6950	0.7496	0.6827
Sichuan	0.6427	0.6474	0.6439	0.6711	0.6990	0.7102	0.7445	0.6798
Guizhou	0.6303	0.6516	0.6554	0.6579	0.6864	0.6854	0.7127	0.6685
Yunnan	0.6332	0.6140	0.6196	0.6435	0.6687	0.6688	0.7158	0.6519
Shaanxi	0.6674	0.6686	0.6487	0.6796	0.7139	0.7242	0.7251	0.6896
Gansu	0.6327	0.6221	0.5991	0.6295	0.6252	0.6686	0.6546	0.6331
Qinghai	0.6956	0.6982	0.6951	0.7184	0.7100	0.7420	0.7443	0.7148
Ningxia	0.6294	0.6321	0.6458	0.6652	0.6716	0.6770	0.6685	0.6557
Xinjiang	0.6886	0.6896	0.6702	0.6889	0.7427	0.7523	0.7627	0.7136
National	0.6395	0.6437	0.6428	0.6645	0.6742	0.6898	0.7029	0.6653

**Table 5:** P for 30 provinces, 2013–2019.

Region	2013	2014	2015	2016	2017	2018	2019
Beijing	0.7803	0.7820	0.7821	0.7823	0.7827	0.7823	0.7834
Tianjin	0.7395	0.7744	0.7811	0.7823	0.7827	0.7685	0.7780
Hebei	0.6012	0.6350	0.5531	0.7833	0.7935	0.7969	0.7983
Shanxi	0.4255	0.4515	0.4631	0.6298	0.6610	0.6716	0.6492
Inner Mongolia	0.6375	0.6490	0.6622	0.7778	0.7161	0.6500	0.7397
Liaoning	0.7333	0.6773	0.7048	0.8255	0.7987	0.7947	0.7206
Jilin	0.7698	0.7682	0.7867	0.9334	0.9317	0.9363	0.9247
Heilongjiang	0.6528	0.6577	0.6623	0.8830	0.8917	0.9025	0.9004
Shanghai	0.7761	0.7820	0.7821	0.7823	0.7827	0.7823	0.7834
Jiangsu	0.7588	0.7762	0.7866	0.8094	0.7841	0.7847	0.7835
Zhejiang	0.8864	0.9054	0.9178	0.9325	0.9232	0.9204	0.9271
Anhui	0.6727	0.7472	0.7748	0.9052	0.8984	0.9031	0.8740
Fujian	0.8408	0.8241	0.9152	0.9682	0.9494	0.9375	0.9414
Jiangxi	0.7013	0.7379	0.7909	0.9189	0.9412	0.9392	0.9533
Shandong	0.7508	0.7378	0.7519	0.7957	0.8057	0.8390	0.7859
Henan	0.6503	0.7273	0.7505	0.8692	0.8896	0.8696	0.8324
Hubei	0.7884	0.8246	0.8609	0.9530	0.9445	0.9296	0.9122
Hunan	0.7707	0.8109	0.8376	0.9719	0.9698	0.9595	0.9700
Guangdong	0.8757	0.8828	0.8909	0.9208	0.9093	0.9165	0.9100
Guangxi	0.7650	0.7988	0.8418	0.9539	0.9621	0.9538	0.8582
Hainan	0.7552	0.7372	0.7129	0.9680	0.9279	0.9111	0.9021
Chongqing	0.8243	0.8390	0.8862	0.9602	0.9619	0.9410	0.9285
Sichuan	0.8118	0.8384	0.8632	0.9723	0.9725	0.9743	0.9739
Guizhou	0.5760	0.7001	0.7684	0.8872	0.9054	0.9101	0.9262
Yunnan	0.6778	0.7709	0.8071	0.9039	0.9339	0.9363	0.9318
Shaanxi	0.7731	0.7906	0.7905	0.8941	0.9148	0.9128	0.8634
Gansu	0.5627	0.5919	0.5610	0.8254	0.8057	0.8518	0.8993
Qinghai	0.5454	0.6145	0.6848	0.8327	0.8421	0.8303	0.8510
Ningxia	0.2686	0.2933	0.2190	0.5892	0.5102	0.5254	0.5281
Xinjiang	0.3352	0.3891	0.5259	0.7152	0.7043	0.8111	0.7733
National	0.6902	0.7172	0.7372	0.8576	0.8532	0.8547	0.8468

## Data Availability

The data presented in this study are available in the article.
